# Rapid and Low-Cost Detection of Millet Quality by Miniature Near-Infrared Spectroscopy and Iteratively Retaining Informative Variables

**DOI:** 10.3390/foods11131841

**Published:** 2022-06-22

**Authors:** Fuxiang Wang, Chunguang Wang, Shiyong Song

**Affiliations:** 1 School of Mechanical and Electrical Engineering, Inner Mongolia Agricultural University, Hohhot 010000, China; fxiangwang@163.com; 2 Mongolia Lvtao Detection Technology Company Limited, Hohhot 010000, China; songshiyong880606@163.com

**Keywords:** miniature near-infrared spectroscopy, foxtail millet, fat content, prediction model

## Abstract

Traditional chemical methods for testing the fat content of millet, a widely consumed grain, are time-consuming and costly. In this study, we developed a low-cost and rapid method for fat detection and quantification in millet. A miniature NIR spectrometer connected to a smartphone was used to collect spectral data from millet samples of different origins. The standard normal variate (SNV) and first derivative (1D) methods were used to preprocess spectral signals. Variable selection methods, including bootstrapping soft shrinkage (BOSS), the variable iterative space shrinkage approach (VISSA), iteratively retaining informative variables (IRIV), iteratively variable subset optimization (IVSO), and competitive adaptive reweighted sampling (CARS), were used to select characteristic wavelengths. The partial least squares regression (PLSR) algorithm was employed to develop the regression models aimed at predicting the fat content in millet. The results showed that the proposed 1D-IRIV-PLSR model achieved optimal accuracy for fat detection, with a correlation coefficient for prediction (Rp) of 0.953, a root mean square error for prediction (RMSEP) of 0.301 g/100 g, and a residual predictive deviation (RPD) of 3.225, by using only 18 characteristic wavelengths. This result highlights the feasibility of using this low-cost and high-portability assessment tool for millet quality testing, which provides an optional solution for in situ inspection of millet quality in different scenarios, such as production lines or sales stores.

## 1. Introduction

Foxtail millet (*Setaria italica*) is a cereal with a long history of cultivation, mainly in the northern regions of China. Millet is grown in a wide range of regions, with China accounting for 80% of the world’s total planted area and an annual production of about 4.5 million tons. It is widely consumed in China because of its rich nutritional content that includes protein, carbohydrates and fats. Typically, fat represents 2–10% of the total weight of millet, depending on the variety and cultivation conditions [1,2]. Accurate detection and quantification of the fat content of millet is important for assessing the nutritional value of millet and therefore usually affects the selling price. Several methods are available for analyzing the fat content of food or agricultural products. One of the most common analytical methods, Soxhlet extraction, is based on determining the weight of crude fat followed by solvent extraction [3]. Other methods of fat analysis are based on acid hydrolysis followed by saponification and esterification of the fat to obtain methyl esters of fatty acids, which can be analyzed by gas chromatography. These methods are accurate but involve analytical processes that are time-consuming and often involve a variety of high-cost analytical instruments, such as gas chromatography–mass spectrometry. The analysis process also involves the use of various chemical reagents that are potentially biotoxic and environmental contaminants. These destructive analytical methods preclude further analysis or processing of samples in a batch.

Near-infrared spectroscopy (NIRS) is a well-established detection technique with the advantages of being green, nonpolluting, rapid and nondestructive and is therefore suitable for large-batch and high-throughput sample analysis. The NIR spectral region lies between 780 and 2526 nm, the wavelength range of the overtones and combinations of vibrations of hydrogen-containing groups (for example, C–H, O–H, and N–H) [4,5]. The NIR spectral signal of a test sample can be combined with chemometric algorithms to analyze the composition. Numerous studies have confirmed the feasibility of NIR for quantifying the chemical composition of cereals or agricultural products. For example, the moisture, ash, protein, lipid and carbohydrate composition of Brazilian soybean seeds were successfully predicted by Fourier transform near-infrared spectroscopy (FT-NIRS) [6]. The protein, dietary fiber and fatty acid contents of marama beans cultivated in different regions were estimated by comparative spectroscopy, including NIRS [7]. Other NIRS-based applications include the determination of the main chemical constituents in *Chenopodium quinoa* grain [8] and the fermentation index, pH and polyphenols in cocoa beans [9,10]. Studies have also been performed to determine the fat content of cereals. Bilal et al. determined the fat content of peanut seeds by using portable near-infrared spectroscopy, extending the use of this technology to the fast and nondestructive screening of quality parameters of peanut seeds [11]. Teye et al. used FT-NIRS to determine the fat content of cocoa beans. Accurate predictions have been made using synergy interval support vector machine regression (Si-SVR) models combined with efficient spectral variable selection, with a root-mean-square error (RMSE) of 0.015 and a correlation coefficient (R) of 0.970 for the prediction set [12]. Despite these advances, the feasibility of NIRS for assessing quality parameters in millet samples, an ancient Chinese grain, has not been reported.

The configuration of a spectroscopic instrument is crucial for obtaining a clear spectral signal. A benchtop Fourier NIR spectrometer is usually used to obtain a high-quality spectral signal. However, benchtop NIR instruments have drawbacks. For example, these instruments usually have a high commercial price, which makes it difficult for NIRS-based detection techniques to become a routine analytical tool. In addition, benchtop NIR spectrometers have poor portability, making it difficult to evaluate samples effectively and in situ in scenarios outside the laboratory, such as manufacturing and sales. Fortunately, the advent of small or miniature NIR spectrometers has enabled an increasing number of researchers to use smartphone- or tablet-connected miniature NIR spectrometers for the rapid determination of the quality of agricultural products [13,14,15]. Micro-NIR spectrometers have been shown to achieve assessment accuracies similar to those of benchtop NIR instruments [16,17]. More importantly, miniature NIR spectrometers are cost-effective, small and portable, and therefore suitable for in situ detection without location constraints.

Thus, the aim of this study was to investigate the potential of smartphone-connected micro-NIR spectroscopy to quantify the fat content of millet. The results of this study can provide a reference for rapid, low-cost and in situ detection of the quality parameters of millet, as well as other agricultural products, and promote scientific evaluation and effective control of agricultural product quality.

## 2. Materials and Methods

### 2.1. Sample Preparation

**Figure 1:**
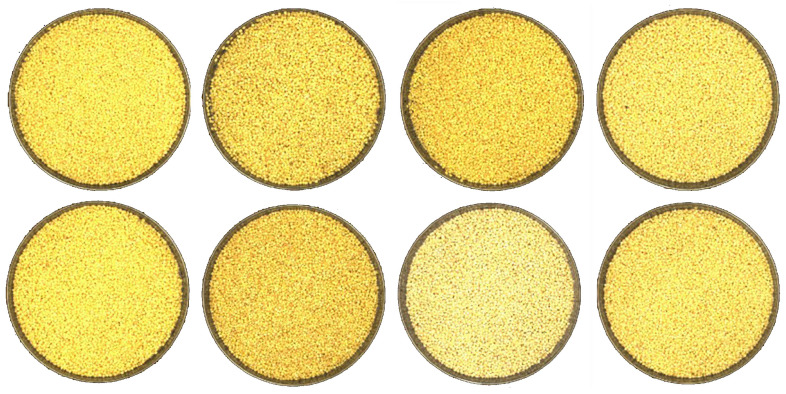
Representative millet samples used in the study.

The millet samples used in this study were from Inner Mongolia, a major millet-producing province in China. To increase sample variability, millet samples were collected from ten farms in four different regions of Inner Mongolia, including Chifeng, Bayannur League, Hohhot, and Hinggan League (Figure 1). Thirty samples were collected from each region, corresponding to a total of 120 millet samples. The samples were sealed in the laboratory immediately upon receipt and stored in a 4-degree refrigerator until analysis.

### 2.2. NIR Spectral Data Collection

Miniature NIR spectrometers are preferred by researchers for their tiny size, low price and high portability. In this study, we used a miniature NIR spectrometer (model NIR-S-R2, InnoSpectra Corporation, Taiwan, China). This ultraportable NIR spectrometer is based on a digital light processing (DLP) technique developed by Texas Instruments and is equipped with an InGaAs detector for high-performance measurements. The wavelength range of the micro-NIR is 900–1700 nm, with 228 continuous wavelengths (spectra resolution of 3.51 nm). The instrument is extremely compact, with a length, width and height of 75, 58 and 26 mm, respectively, and weighs 77 g. In this study, dedicated spectrum-acquisition software was installed on a smartphone according to the manufacturer’s instructions. The smartphone was connected to the NIR spectrometer via Bluetooth and used for parameter setting, data acquisition and storage. The samples were prepared for data collection by being placed in a circular petri dish and weighed to approximately 10 g. The NIR spectrometer was then inverted and placed in contact with the millet sample, with the detection port oriented toward the millet sample. Data were collected at 5 random points in the millet sample, and the average of these 5 scans was subsequently used as representative sample data. The scanned data were stored in .csv format in the smartphone and subsequently imported to a computer for analysis.

### 2.3. Fat Content Measurement

After spectra collection, all millet samples were freeze-dried to remove moisture. The dried millet was ground into powder. The fat content of the millet was determined according to the Chinese National Standard: GB 5009.6-2016 (National Standard for Food Safety—Determination of fat in food). Specifically, approximately 2–5 g (0.001 g) of the powdered millet sample was weighed and placed in a 50-mL test tube. Then, 8 mL of water and 10 mL of hydrochloric acid were added to the tube. The test tube was placed in a water bath at 70–80 °C and stirred with a glass rod until the sample was completely digested, corresponding to a period of approximately 40–50 min. The tube was removed from the bath, and 10 mL of ethanol and 25 mL of anhydrous ether were added to the tube. The tube was allowed to stand for 10–20 min; when the upper liquid was clear, the supernatant was aspirated in a constant-weight conical flask, and 5 mL of anhydrous ether were added to a measuring cylinder with a stopper. The liquid was left to stand, and the upper liquid layer was sucked out and placed in the original conical flask. The sample was dried at a temperature of 10 ± 5 °C for 1 h, cooled in a desiccator for 0.5 h and then weighed. The aforementioned operation was repeated until a constant weight was measured for the sample. Each sample was tested three times, and the average result was taken as the fat content of the sample. In general, increasing the content span of the samples in the calibration set improves the robustness of the developed model.

### 2.4. PLSR Models Development

#### 2.4.1. Dataset Split

Before modeling, sample data must be divided into calibration and prediction sample sets. The calibration set is used to develop a prediction model that is verified using the prediction set. Several methods are commonly used to partition the dataset, including random division, sorting division, and Kennard–Stone. Sorting division was used in this study, being a simple method that ensures that the content span of the samples in the prediction set is covered by the calibration set samples. The division procedure was as follows: all samples were ranked from lowest to highest in terms of the fat content. Then, the middle sample of every three samples was assigned to the prediction set, and the remaining two samples were assigned to the calibration set, resulting in a ratio of 2:1, that is, 80 samples were classified into the calibration set, and the remaining 40 samples were grouped into the prediction set.

#### 2.4.2. Spectral Preprocessing

The obtained raw spectral information usually contains interference information, such as noise, which degrades the information quality. Therefore, spectral preprocessing is essential. Here, standard normal variate (SNV) and first derivative (1D) with Savitzky–Golay methods were used to preprocess the spectral signals. SNV is used to eliminate the interference caused by the size of solid particles, surface scattering and light range variations in acquired spectra [18]. 1D eliminates the baseline shift and separates broad and overlapping NIR bands in spectra without increasing the noise [19].

#### 2.4.3. PLSR Modeling

The partial least squares regression (PLSR) model is a commonly used linear modeling tool. PLSR linearly transforms raw data into a new database of variables called latent variables (LVs). The LVs are usually optimized by analyzing the prediction residual error sum of squares (PRESS) curve obtained from Monte Carlo validation [20]. The number of LVs corresponding to the lowest PRESS is the optimal LV. In this study, the PLSR model was established considering both the independent variable (the NIR spectral data) and the dependent variable (the fat content). Data from all wavelengths in the complete spectrum were used for modeling. For feature selection, only the spectral data at the selected wavelengths were extracted and used to build the PLSR model.

The performance of the developed PLSR models was assessed using the following parameters: the correlation coefficients for calibration (Rc) and prediction (Rp), root mean square errors for calibration (RMSEC) and prediction (RMSEP), and residual predictive deviation (RPD). In addition, the ratio of Rc to Rp was used to evaluate the stability and robustness of the proposed model.

#### 2.4.4. Characteristic Wavelength Selection

The NIR spectral data obtained at 228 wavelengths includes redundant information that makes the modeling process complex and inefficient. Additionally, spectral signals at different wavelengths have different correlations with target compounds. Therefore, several wavelength selection methods, including bootstrapping soft shrinkage (BOSS), the variable iterative space shrinkage approach (VISSA), iteratively retaining informative variables (IRIV), iteratively variable subset optimization (IVSO), and competitive adaptive reweighted sampling (CARS), are commonly used to select the characteristic wavelengths that contribute significantly to the fat content to be detected.

The BOSS method is used in conjunction with the PLSR model [21]. This method is designed to select collinear information variables. This method utilizes the information from the regression coefficient (RC) of the PLSR model in conjunction with an effective method for soft shrinkage. In BOSS, bootstrap sampling and weighted bootstrap sampling (WBS) functions are used to generate random combinations of variables and construct submodels, from which information is extracted by model population analysis (MPA).

VISSA is based on the concept of MPA and used to select features in spectral variables [22]. Unlike existing methods for variable selection optimization, VISSA statistically evaluates the performance of the variable space at each optimization step. WBM sampling is used to build submodels across the subspace of variables. Two rules are followed during the optimization process. First, the variable space is reduced at each step. Second, the new variable space is preferred over the previous space. The second rule, which is rarely satisfied in most existing methods, is the core of the VISSA strategy.

IRIV is a newly proposed variable selection method in which BMS is used to generate a large number of different combinations of variables in the variable space [23]. For each variable, IRIV determines the difference between the root mean square errors for cross-validation (RMSECV) of all variable combinations with and without the variable. An increase in the RMSECV when the considered variable is excluded from the combination of variables indicates that the variable is useful. The converse result indicates that the variable is not useful. The IRIV iteratively retains informative variables until no uninformative or intrusive variables remain, and the 10-fold cross-validation was used to determinate the RMSECV.

IVSO was proposed in recent years for NIR spectral variable screening [24]. In IVSO, the weighted binary matrix sampling (WBMS) method is introduced for random sampling and elimination of spectral variables with small contributions. The weight values of all spectral variables would be evaluated and sorted. If the weight value of a spectral variable is small, then the probability that this variable is selected is low; the variable may not be selected. In this method, a wavelength with a smaller weight has a lower probability of being selected. A two-step procedure, including WBMS and sequential addition, is used to gradually eliminate nonreference variables in a competitive manner and reduce the risk of losing key variables.

CARS is one of the most widely used methods for spectral selection [25]. In CARS, the absolute value of the regression coefficients of the developed PLSR model is used as an indicator of the significance of each variable. The subset of spectral variables is selected sequentially using Monte Carlo resampling in an iterative and competitive manner. In this study, the optimal subset of spectral variables was determined based on the minimum RMSECV. In each CARS sampling run, 80% of the samples in the calibration set were randomly selected to build the PLSR model.

## 3. Results and Discussion

### 3.1. Crude Fat Content of Millet

The fat content of the millet samples was determined by traditional chemical analysis, and the results are shown in Table 1. Specifically, the fat content of all 120 samples ranged from 2.84–6.03 g/100 g, with a mean value of 4.42 g/100 g. This range value was higher than the content of 3.75–5.34 g/100 g reported by Feng et al., [1] and 4.0–4.5 g/100 g reported by Yang et al., [2]. Possible reasons for this discrepancy are differences in varieties and cultivation practices of the investigated samples [2]. In this study, millet samples of different varieties and from different farms were collected and analyzed to develop a predictive model with wide applicability. The fat contents in the calibration and prediction sets were 2.84–6.03 g/100 g and 2.87–6.02 g/100 g, respectively, showing that the content span of the prediction set samples was covered by the calibration set.

### 3.2. Spectral Features of Millet

**Figure 2:**
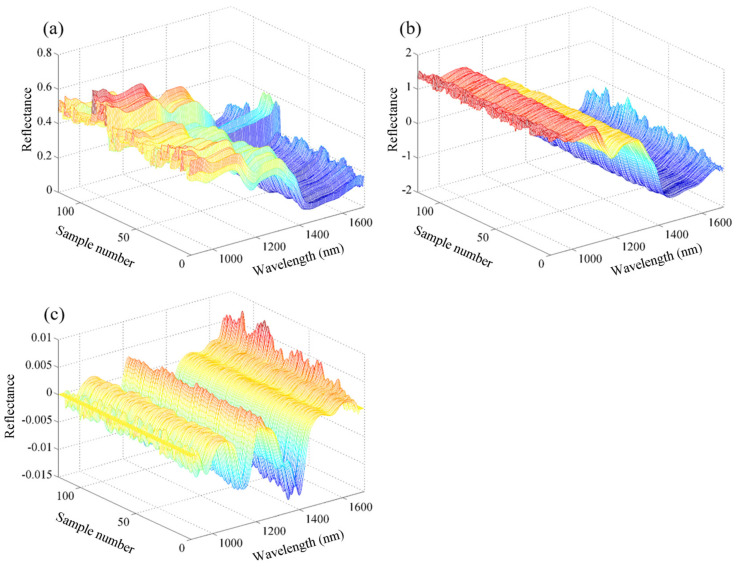
Spectral curves of all millet samples by using (**a**) raw data; (**b**) SNV preprocessed data; and (**c**) 1D preprocessed data.

The raw spectral profiles of all the millet samples are shown in Figure 2a. The spectral trends of all the samples were similar, i.e., there were clear absorption peaks at 960–980 nm, 1190–1210 nm, and 1440–1480 nm. These peaks are representative of the functional groups of the chemical components of the millet samples. The absorption peak appearing in the 960–980 nm range may be related to the second O–H overtone of water [15]. Esteban-Diez et al. attributed the absorption band at 1208 nm to the C–H stretching second overtone (–CH_3_ or –CH_2_) of carbohydrates [26]. Osborne et al. assigned the absorption band at 1202 nm to the second overtone of O–H stretching in starch [27]. He et al. assigned the band at 1210 nm to the second overtone of C–H stretching in fat [28]. Thus, the absorption peak at 1190–1210 nm may be attributed to the presence of carbohydrates, starch, and fat. The bands at approximately 1440 nm may be attributed to the first overtone of the O–H stretching of water [29] and the first overtone of the N–H stretching vibrations of proteins [30].

### 3.3. Optimization of Spectral Preprocessing

**Figure 3:**
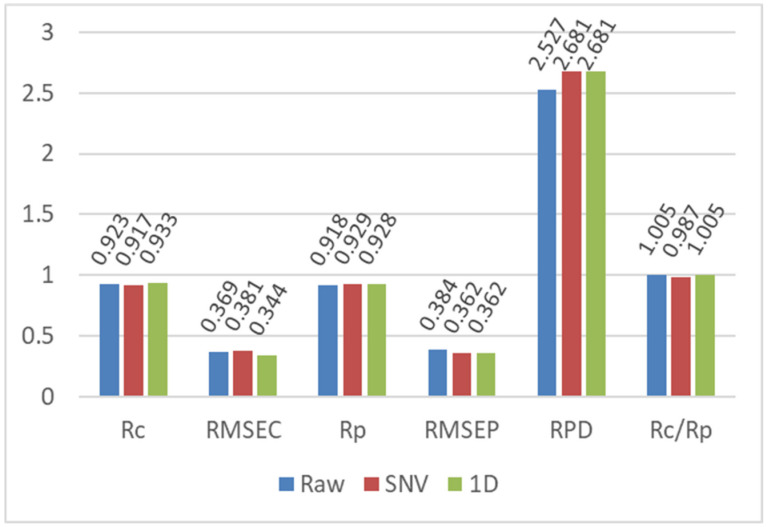
Results of PLSR prediction models for the fat content based on the raw spectra and spectra preprocessed by SNV and 1D.

Figure 2b,c shows the spectral curves of millet samples preprocessed by SNV and 1D, respectively. The preprocessed spectral curves were smoother than the raw spectral curves. To compare the performance of different preprocessing methods in removing irrelevant information, such as noise, PLSR prediction models for the fat content were developed based on the raw spectra and spectra preprocessed by SNV and 1D, and the results are shown in Figure 3. The PLSR model based on the raw spectra exhibited good predictive accuracy with an Rc value of 0.923, RMSEC of 0.369 g/100 g, Rp of 0.918, RMSEP of 0.384 g/100 g and RPD value of 2.527. The RPD is an important indicator of the accuracy of a developed model. RPDs over 2.0 and 3.0 indicate good and excellent model accuracy, respectively. The ratio between Rc and Rp indicates the stability of a model. The closer the ratio is to 1, the more stable the model is. Therefore, the PLSR model based on the raw data had good prediction performance and high stability. The different preprocessing methods improved the performance of the developed PLSR models to different degrees, where higher Rp and RPD values and lower RMSEP values were obtained using both SNV and 1D. This result indicates that the noise in the raw spectral information can be reduced or eliminated by preprocessing. For the prediction set, the SNV and 1D achieved equivalent performances with an Rp of 0.928, RMSEP of 0.362 g/100 g and RPD of 2.681. By contrast, the 1D-based PLSR model had an Rc/Rp of 1.005, which was better than that of the SNV-based model of 0.987. Thus, 1D was the more suitable preprocessing method for fat content determination.

### 3.4. Prediction Models Based on Characteristic Wavelengths

**Figure 4:**
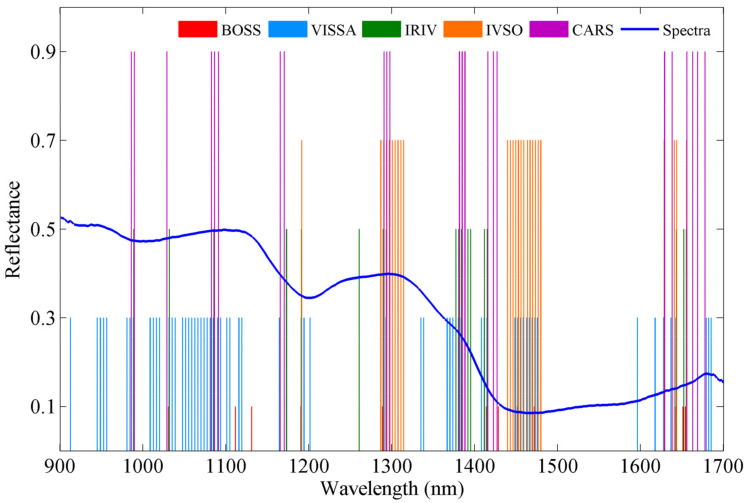
Characteristic wavelengths selected by BOSS, VISSA, IRIV, IVSO and CARS.

BOSS, VISSA, IRIV, IVSO and CARS were used to select characteristic wavelengths from the complete set of wavelengths, and the results are shown in Figure 4. In summary, 12, 65, 18, 27 and 23 characteristic wavelengths were selected by BOSS, VISSA, IRIV, IVSO and CARS, respectively. Wavelength selection significantly reduced the total number of spectral variables by 71.49–94.74%. These results show the advantages of dimensionality reduction, although different methods produced different results.

**Figure 5:**
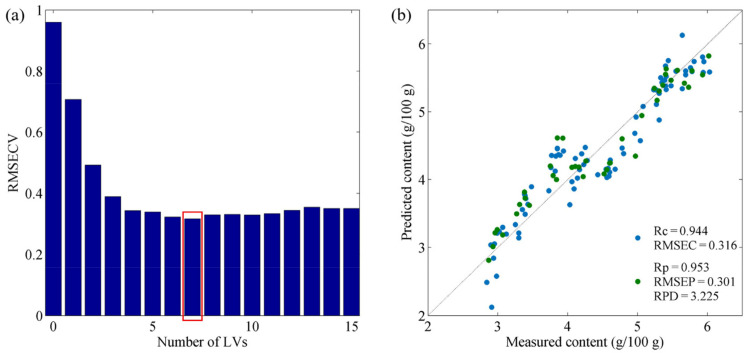
Plots of the optimal IRIV–PLSR model: (**a**) selection of the optimal number of LVs, and (**b**) scatter plot of the IRIV–PLSR model for fat prediction.

The PLSR models established using the selected wavelengths are denoted as BOSS–PLSR, VISSA–PLSR, IRIV–PLSR, IVSO–PLSR and CARS–PLSR. The prediction results of all the models are shown in Table 2. For the calibration set, all models achieved comparable performance to that of the PLSR model based on the complete spectrum, with Rc values in the range of 0.93–0.95 and RMSEC values of 0.31–0.36 g/100 g. When the developed models were validated by the prediction set, all the models outperformed the PLSR model based on the complete spectrum, with higher Rp and RPD values and lower RMSEP values. Among the five aforementioned PLSR models, BOSS–PLSR, VISSA–PLSR, IRIV–PLSR and CARS–PLSR outperformed the PLSR model based on the complete spectrum and had RPD values over 3.0, indicating the excellent accuracies of the developed models. Notably, the IRIV–PLSR model exhibited the optimal predictive performance, with an Rp of 0.953, RMSEP of 0.301 g/100 g and RPD of 3.225, based on selecting 18 characteristic wavelengths and 7 LVs (Figure 5a). The 18 selected wavelengths were as follows: 988, 1031, 1085, 1173, 1191, 1260, 1289, 1293, 1377, 1380, 1384, 1391, 1395, 1411, 1415, 1643, 1652 and 1654 nm. In addition, the IRIV–PLSR model had an Rc/Rp of 0.991, showing the model was stable. Scatter plots of the predicted fat content by the best model (the IRIV–PLSR) are shown in Figure 5b; the measured fat contents are very close to the model predictions, indicating the excellent predictive performance of the IRIV–PLSR model.

### 3.5. Discussion

**Figure 6:**
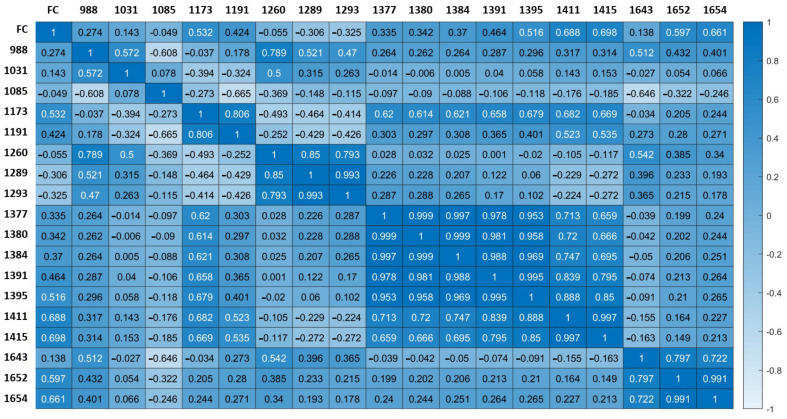
Heat map of the correlation coefficient between the selected characteristic wavelengths by IRIV and fat content.

Near-infrared spectroscopy is a well-established detection technique for the analysis of agricultural components. Spectral variable selection is a key aspect of NIR data processing that is important for data dimensionality reduction and the optimization of model performance. In this study, we evaluated the potential of traditional CARS and several emerging methods, including BOSS, VISSA, IRIV, and IVSO, for selecting characteristic wavelengths for fat. Our results show the advantages of variable selection, with models based on all wavelength selection methods outperforming the model based on the complete spectrum, as evidenced by higher RPD values and lower RMSEP values. Among the considered wavelength selection methods, IRIV exhibited the best predictive performance while reducing the number of wavelengths to 18. The reason for this excellent performance may be that IRIV uses a large number of different combinations of variables to evaluate the importance of each variable during each round of the run. This process requires considerable computational time but is effective in eliminating irrelevant or low-importance spectral variables. The correlation between each wavelength selected by IRIV method and the fat content was analyzed, and the results are shown in Figure 6. The spectral data at 1173, 1395, 1411, 1415, 1652 and 1654 nm showed a strong positive correlation with the fat content, with correlation coefficients over 0.5. The selection of these wavelengths has a positive effect on the next step in the development of filter-based optical sensors.

The feasibility of NIRS in predicting the fat content of millet has not been previously explored. Nevertheless, some progress has been made in the detection of the fat content of other agricultural products by NIRS. Bilal et al. used NIRS for determining the fat content of peanut seeds, where the optimal model performance corresponded to 0.9388, 0.47 and 3.40 for the Rp, RMSEP and RPD, respectively [11]. Teye et al. employed FT-NIRS to determine the fat content of cocoa beans. Using synergy interval support vector machine regression (Si-SVR) models combined with efficient spectral variable selection resulted in accurate prediction with an RMSEP of 0.015 and an Rc of 0.970 [12]. The optimal model constructed in this study has an Rp of 0.953 and RPD of 3.225, which is comparable to the aforementioned results. However, the model constructed in this study has more applications than those based on the benchtop NIRS used in most studies [12]. The NIR spectrometer used in this study is a miniature instrument, which has the advantages of a low cost and high portability. Additionally, a smartphone app could be developed based on the built model. Connecting this inexpensive optical sensor to a smartphone provides a novel solution for on-site fat assessment of millet in most situations, such as production lines or sales stores.

## 4. Conclusions

In this study, we evaluated the fat content of millet with different origins using smartphone-connected micro-NIR spectroscopy. Spectral preprocessing and variable selection methods were compared and optimized, and the best prediction model for the fat content was determined. The optimal 1D-IRIV-PLSR model had accurate predictive performance with an Rp of 0.953, RMSEP of 0.301 g/100 g and RPD of 3.225 while using only 18 characteristic wavelengths. The results highlight the feasibility of this low-cost and high-portability assessment tool for millet quality testing. The high-portability tool utilized in the study should be used to develop specialized analysis software for smartphones and linked to low-cost test equipment to enable rapid and on-site testing of millet quality.

## Figures and Tables

**Table 1:** Statistical analyses of measured fat contents in all millet samples in the calibration and prediction sets.

Dataset	N	Range (g/100 g)		Mean	SD	C.V (%)
		Min	Max			
Calibration	80	2.84	6.03	4.42	0.97	21.95
Prediction	40	2.87	6.02	4.42	0.96	21.77
All	120	2.84	6.03	4.42	0.96	21.74

N: number of samples; SD: standard deviation; C.V: coefficient of variance—the ratio of the mean value to SD.

**Table 2:** Performance of PLSR models with different wavelength selection approaches for the prediction of fat content.

Method	NVs	LVs	Calibration Set		Prediction Set			Rc/Rp
			Rc	RMSEC	Rp	RMSEP	RPD	
None	228	8	0.933	0.344	0.928	0.362	2.681	1.005
BOSS	12	5	0.931	0.351	0.943	0.321	3.024	0.987
VISSA	65	6	0.939	0.329	0.948	0.316	3.072	0.991
IRIV	18	7	0.944	0.316	0.953	0.301	3.225	0.991
IVSO	27	4	0.933	0.345	0.941	0.331	2.932	0.991
CARS	23	6	0.936	0.336	0.949	0.311	3.121	0.986

## Data Availability

The data presented in this study are available on request from the corresponding author.
